# Dioxin-Induced Changes in Epididymal Sperm Count and Spermatogenesis

**DOI:** 10.1289/ehp.0901084

**Published:** 2009-12-17

**Authors:** Warren G. Foster, Serena Maharaj-Briceño, Daniel G. Cyr

**Affiliations:** 1 Reproductive Biology Division, Department of Obstetrics and Gynecology, McMaster University, Hamilton, Ontario, Canada; 2 INRS-Institut Armand-Frappier, Université du Québec, Laval, Quebec, Canada

**Keywords:** development, dioxin, endocrine, reproduction, spermatogenesis

## Abstract

**Background:**

A single *in utero* exposure to 2,3,7,8-tetrachlorodibenzo-*p*-dioxin (TCDD) on gestation day 15 decreased epididymal sperm count in adult rats and thus was used to establish a tolerable daily intake for TCDD. However, several laboratories have been unable to replicate these findings. Moreover, conflicting reports of TCDD effects on daily sperm production suggest that spermatogenesis may not be as sensitive to the adverse effects of TCDD as previously thought.

**Data sources:**

We performed a PubMed search using relevant search terms linking dioxin exposure with adverse effects on reproduction and spermatogenesis.

**Data synthesis:**

Developmental exposure to TCDD is consistently linked with decreased cauda epididymal sperm counts in animal studies, although at higher dose levels than those used in some earlier studies. However, the evidence linking *in utero* TCDD exposure and spermatogenesis is not convincing.

**Conclusions:**

Animal studies provide clear evidence of an adverse effect of *in utero* TCDD exposure on epididymal sperm count but do not support the conclusion that spermatogenesis is adversely affected. The mechanisms underlying decreased epididymal sperm count are unknown; however, we postulate that epididymal function is the key target for the adverse effects of TCDD.

Dioxins are lipophilic chemicals that resist biological and environmental degradation, making them persistent in the environment. Seventy-five dioxin congeners and 135 furan congeners comprise the complex mixture of dioxins, of which 7 and 10 congeners, respectively, are capable of binding to and activating the aryl hydrocarbon receptor (AhR) (Van den Berg et al. 2006). Of the 209 polychlorinated biphenyl (PCB) congeners, 12 have the potential to activate the AhR (Van den Berg et al. 2006). Among these, 2,3,7,8-tetrachlorodibenzo-*p*-dioxin (TCDD) is the most toxic environmental contaminant in animal studies (Denison and Nagy 2003) and thus is significant for human health (Birnbaum 1994; Larsen 2006; Schecter et al. 2006).

Dioxins are by-products of industrial processes such as chlorine bleaching of pulp and paper, the manufacture of certain pesticides, and incineration of medical waste and plastics (Anderson and Fisher 2002; Hewitt et al. 2006; Lin et al. 2006; Thornton et al. 1996). Resistance to degradation leads to bioaccumulation and biomagnification of dioxins in the food chain. Inclusion of animal fat in animal feed is another route of dioxin entry to the food supply and a source of exposure (Hoogenboom et al. 2007). Human exposure is primarily through consumption of contaminated food, especially high-fat foods such as milk, cheese, meat, some fish, fast foods, and breast milk (Schecter and Li 1997; Schecter et al. 1997, 1998a, 1998b, 2001). Residue levels have been measured in the serum of pregnant Canadian women with a mean ± SE of 0.34 ± 0.01 pg TCDD/g lipid (Foster et al. 2005), lower than the serum concentrations measured in pregnant German women (range, 4.34–97.3 pg TCDD/g lipid; Wittsiepe et al. 2007) and women from central Taiwan (mean, 6.7 pg TCDD/g lipid; Wang et al. 2004). The different concentrations in these studies reflect differences in measurement techniques; a gene reporter assay [chemical-activated luciferase gene expression (CALUX) assay] and high- resolution mass spectrometry have been used to quantify World Health Organization (WHO) toxic equivalence quotient concentrations or dioxin-like activity. Regardless, these studies demonstrate that the fetus is exposed to dioxin-like chemicals during a critical window of development. The half-life of dioxin ranges from 5.8 to 14.1 years in humans and is influenced by body composition, with higher body fat associated with a longer half-life (Michalek et al. 1992, 1996, 2002). By comparison, the half-life ranges from 10 to 15 days in mice (Grassman et al. 1998) and is approximately 3 weeks in rats (Rose et al. 1976). Given the documented adverse effects on the adult rat reproductive tract after a single *in utero* exposure to TCDD, developmental exposure and differences in the half-life of TCDD could have important consequences for the relevance of results from animal studies for human health.

Experimental evidence demonstrates that most toxic actions of TCDD are mediated through the AhR, which is a ligand-activated transcription factor (Robles et al. 2000) ubiquitously expressed in many human tissues and cell lines (Dolwick et al. 1993; Harper et al. 1991; Li et al. 1998). AhR is inactive and unbound in the cytoplasm. Upon ligand binding, the AhR binds to the aryl hydrocarbon nuclear translocator (ARNT) protein, resulting in translocation to the nucleus, where the ligand–AhR–ARNT complex binds to response elements in the promoter of AhR-regulated genes (dioxin response element).

In humans, exposure to dioxins has been linked to a variety of adverse effects, including chloracne (Baccarelli et al. 2005a, 2005b), immune suppression (Weisglas-Kuperus et al. 2000), thyroid dysfunction (Koopman-Esseboom et al. 1994; Pavuk et al. 2003), increased risk for diabetes (Longnecker and Michalek 2000) and endometriosis (Eskenazi et al. 2000; Heilier et al. 2007), impaired neurodevelopment (Koopman-Esseboom et al. 1996; Vreugdenhil et al. 2002), and reproductive/developmental abnormalities (Dimich-Ward et al. 1996; Halldorsson et al. 2009; Leijs et al. 2008; Mocarelli et al. 1996, 2000, 2008). A number of studies attribute background dioxin exposure to adverse effects on development or pathophysiology in multiple organ systems. However, the results of these studies are controversial, and it is not possible to establish causal associations; thus, animal studies are essential.

In animal studies, dioxin exposure has been shown to cause thymic atrophy (Chahoud et al. 1989), immune suppression (Hogaboam et al. 2008), hepatotoxicity (Chahoud et al. 1989), and impaired thyroid function (Fan and Rozman 1995; Henry and Gasiewicz 1987; Kohn 2000). Of these, TCDD effects on the reproductive tract are the most notable, owing to the sensitivity of this system. Developmental exposure to TCDD induces placental dysfunction (Ishimura et al. 2006; Kawakami et al. 2006); decreased offspring survival (Bell et al. 2007a, 2007b; Bjerke and Peterson 1994; Flaws et al. 1997; Gray and Ostby 1995; Gray et al. 1995, 1997b; Roman et al. 1995; Sommer et al. 1996); developmental defects of the palate, heart, and kidney (Aragon et al. 2008; Birnbaum et al. 1989; Theobald and Peterson 1997) and reproductive tract of males (Gray et al. 1997a; Vezina et al. 2008) and females (Flaws et al. 1997; Gray and Ostby 1995; Gray et al. 1997b; Heimler et al. 1998; Wolf et al. 1999); decreased weights of reproductive organs (Bjerke and Peterson 1994; Gray and Ostby 1995; Gray et al. 1995, 1997a; Loeffler and Peterson 1999; Mably et al. 1992a, 1992c; Moore et al. 1985; Ohsako et al. 2001, 2002; Wolf et al. 1999); delayed onset of sexual maturation (Bjerke et al. 1994; Faqi and Chahoud 1998; Flaws et al. 1997; Gray et al. 1997a, 1997b); feminization of males (Bjerke et al. 1994; Mably et al. 1992b); and decreased sperm counts (Faqi et al. 1998; Gray et al. 1995, 1997a; Mably et al. 1992a; Sommer et al. 1996). Of the reproductive/developmental effects of TCDD, decreased sperm counts are considered as the most sensitive outcome. Mably et al. (1992a) reported that epididymal sperm counts were significantly decreased in rats after a single exposure to 0.064 μg TCDD/kg on gestational day (GD) 15. Similarities in spermatogenesis between rats and humans, together with the marked apparent sensitivity of sperm production to the adverse effects of TCDD exposure, has yielded the establishment of a tolerable daily intake of approximately 2 pg/kg/day for TCDD and related compounds by the WHO [Joint FAO/WHO (Food and Agriculture Organization/WHO) Expert Committee on Food Additives 2001]. However, results of recent studies have been unable to reproduce the effect of *in utero* TCDD exposure on epididymal sperm counts (Bell et al. 2007a, 2007c; Ohsako et al. 2001; Yonemoto et al. 2005), although one study did report a decrease in epididymal sperm counts with a TCDD concentration of 1 μg/kg (Ohsako et al. 2001). Reasons for the divergent results are unclear, but differences in methodology used to quantify sperm count could account for the observed differences. In addition, disparities in dioxin toxicokinetics and species sensitivity to dioxins raise important questions concerning the use of animal models to estimate human risk (Adler 1996; Aylward et al. 2005; Simanainen et al. 2004a, 2004b). Therefore, the objective of this review is to evaluate the effects of TCDD exposure on spermatogenesis.

We undertook a systematic review of the literature and performed a PubMed (National Center for Biotechnology Information 2009) search using the following search terms: dioxin, TCDD, reproductive, developmental, testis, spermatogenesis, sperm, semen quality, fertility, and fecundity. The search yielded 4,224 titles; duplicate papers, review articles, letters to the editor, and articles describing tissue culture or nonmammalian studies were excluded from further analysis. The abstracts of all remaining articles were read by two independent investigators and were included for further analysis if they fit the following criteria: *a*) the paper was published in English, *b*) the abstract described an epidemiologic or animal study in which TCDD or dioxin-like chemicals were either measured in human tissues or administered to experimental animals; and *c*) effects on reproductive organs, sperm count, and sperm characteristics were assessed. From the original data set, 33 articles described the reproductive toxicity of TCDD; of these, 9 specifically examined the effect of a single *in utero* TCDD exposure on spermatogenesis. For each paper, we extracted details of the experimental methods and the resulting data for further study.

## Dioxin Exposure and Spermatogenesis

Of the reproductive/developmental effects documented in the literature, spermatogenesis is considered the most sensitive adverse effect of TCDD exposure, as shown by the use of this end point by the WHO in setting its tolerable daily intake for TCDD (Joint FAO/WHO Expert Committee on Food Additives 2001). Sperm counts are decreased by up to 36% compared with controls (Bjerke and Peterson 1994), with the lowest effective dose of 0.064 μg TCDD/kg body weight (BW) reported for Holtzman rats (Mably et al. 1992a). GD15 is the most sensitive time point for the adverse effects of TCDD exposure because effects on spermatogenesis in rats with lactational exposure were less pronounced (Bjerke and Peterson 1994). Furthermore, the effects are thought to be AhR mediated because severity of the TCDD effect on epididymal sperm count is modified in rats bearing different resistance alleles (Simanainen et al. 2004a, 2004b).

Although the process of spermatogenesis is qualitatively similar in rats and humans, there are important differences. In rats, spermatogenetic cycles begin every 12.9 days, whereas in humans there are 16 days between cycles; a spermatogenic cycle requires 52–54 days in rats and 65 days in humans to complete (Adler 1996; Hess et al. 1990; Robb et al. 1978). Spermatogonial differentiation lasts 12 days in rats and 16 days in humans (Adler 1996), and development of spermatocytes requires 14 days in rats and 25 days in humans. Spermiogenesis, the process of differentiation of haploid germ cells from round to elongated spermatids, takes another 7–14 days in rats and 8–17 days in humans. Throughout spermatogenesis, genes are differentially expressed in a stage-dependent manner (Pang et al. 2006), and transcripts for *AhR* and *ARNT* have been documented in the rat testes, epididymides, seminal vesicles, vas deferens, and prostate (Roman et al. 1998). In humans, the *AhR* and *ARNT* are expressed in spermatocytes, where they are thought to play a role in regulating apoptosis of spermatocytes (Schultz et al. 2003). Therefore, all of the requisite signaling machinery is present in the testis of both rats and humans. There is limited evidence that developmental exposure to TCDD results in testicular exposure. The highest maternal dose of TCDD used (0.8 μg TCDD/kg BW) in one study resulted in testicular levels of 0.49 pg TCDD/g wet testis on postnatal day (PND) 120, demonstrating that residue levels in target tissue can persist throughout the animal’s life (Ohsako et al. 2001). However, the relevance of animal studies to human risk is still questionable. First, although human exposure to dioxin and dioxin-like chemicals continues to be widespread, tissue residue levels are low relative to the concentrations used in animal studies. Second, the timing of TCDD exposure appears to be critical in establishing the previously documented adverse reproductive phenotype (Mably et al. 1992a). Welsh et al. (2008) suggested a window of programming for the male reproductive tract during embryonic development and described the presence of early (GD15.5–GD17.5), middle (GD17.5–GD19.5), and late (GD19.5–GD21) windows of development, which correspond to the period of development after the onset of fetal androgen production by the rat testis (GD15.5–GD17.5). Subsequent masculinization of the reproductive tract occurs with the morphologic differentiation of the epididymis, vas deferens, seminal vesicles, and prostate, as well as external genitalia (penis, scrotum, and perineum). Fetal androgen development in humans occurs during weeks 8–37 of gestation (Siiteri and Wilson 1974), so the early programming window of development in rats corresponds to weeks 8–14 of development in humans (Welsh et al. 2008). Assuming similar effects in humans, exposure to chemical toxicants, including dioxin, would have to occur during the first trimester of human fetal development to produce a phenotype similar to that observed in rats (Sharpe 2009). However, in humans, the greatest exposure to dioxins occurs during lactation, a period during which rodents are relatively insensitive to the adverse effects of TCDD treatment (Bjerke and Peterson 1994).

The effect of developmental exposure to TCDD on spermatogenesis in rats has raised concerns for potential effects in humans. However, several studies in rats (Bell et al. 2007a; Ohsako et al. 2001; Yonemoto et al. 2005) using similar experimental designs have been unable to replicate the reduction in sperm number reported by Mably et al. (1992a), raising questions concerning the validity of the conclusions and their relevance to human health. Although the reasons are unclear, differences in rat strains cannot account for the divergent results because conflicting results have been documented in all rat strains used to date. It is noteworthy that although several studies have been unable to replicate the findings of Mably et al. (1992a), decreased epididymal sperm counts have been demonstrated at a concentration of 1.0 μg/kg (Ohsako et al. 2001). Others speculate that statistically robust studies employing large sample sizes (25–60 litters/treatment group) are more appropriate and likely to yield more reliable results (Bell et al. 2007a, 2007b). However, effects of TCDD on spermatogenesis have been reported in studies with as few as 3–5 litters (Wilker et al. 1996) to as many as 9–12 litters (Bjerke and Peterson 1994; Gray et al. 1997b; Sommer et al. 1996) per treatment group. Although sample sizes in these studies are small, our calculations demonstrate that to detect a difference of 15 × 10^6^ sperm/mL with an SD of approximately 8.5 × 10^6^, a power of 0.80, and an alpha of 0.5, a sample size of 9 animals/treatment group would be sufficient. Hence, we conclude that most of the reviewed studies were adequately powered and that the documented effects are not likely due to statistical artifact. Rather, we suggest that the divergent results may be due to differences in methods employed to quantify sperm, which have been surprisingly different and range from automated methods to manual counts with a hemocytometer. Indeed, some sperm-counting methodologies can be highly variable, with coefficients of variation as high as 40% as seen in the studies by Bell et al. (2007a, 2007b). Hence, although studies need to be adequately powered, it is also imperative that sensitive outcome measures be employed.

In quantifying sperm count in reproductive/developmental toxicity studies, we propose that there is a need to standardize the methods used, and we favor manual counts with a hemocytometer over automated methods. Furthermore, we found different methods of reporting sperm counts [daily sperm production (DSP)/whole testis (Wilker et al. 1996), DSP/g testis (Wilker et al. 1996), testicular spermatid head count (Gray et al. 1995), and sperm counts from whole epididymis (Bell et al. 2007a, 2007b), caput/corpus epididymis (Gray et al. 1997b; Sommer et al. 1996; Wilker et al. 1996), cauda epididymal sperm (Bjerke and Peterson 1994; Gray et al. 1995; Mably et al. 1992a; Ohsako et al. 2001, 2002; Sommer et al. 1996; Wilker et al. 1996; Yonemoto et al. 2005), and ejaculated sperm (Gray et al. 1995; Sommer et al. 1996)] thus making comparisons difficult. Only two studies (Sommer et al. 1996; Wilker et al. 1996) measured DSP, caput/corpus sperm, and caudal epididymal sperm counts, permitting assessment of potential target sites of TCDD. Interestingly, although both studies agree on the effect of TCDD on caudal epididymal sperm counts, they diverge on the measure of DSP. Furthermore, different time points have been employed to quantify sperm, ranging from PND49, representing puberty (Ohsako et al. 2001; Yonemoto et al. 2005), to 15 months of age (Gray et al. 1995), with most studies quantifying sperm counts between PND63 and PND120, corresponding to postpubertal and adult stages, respectively. Because male rodents are not sexually mature before approximately PND50 and because a spermatogenic cycle takes 52–54 days to complete in the rat (Adler 1996; Robb et al. 1978), measurement of sperm counts in rats before PND70 is likely to produce ambiguous results. Nonetheless, six of eight studies reported a TCDD-induced decrease in cauda epididymal sperm counts (Bjerke and Peterson 1994; Gray et al. 1997b; Mably et al. 1992a; Ohsako et al. 2002; Sommer et al. 1996; Wilker et al. 1996), whereas the evidence for changes in DSP are more variable, with only three of six studies finding a significant decrease (Bjerke and Peterson 1994; Mably et al. 1992a; Sommer et al. 1996). The variable results for DSP are more difficult to interpret because sperm production can be affected by many factors, including nutrition, infection, and general health of the animal, as well as time from last ejaculation and toxicant effects on the hypothalamic–pituitary–testicular axis.

Prior studies have demonstrated treatment-related decreases in BW (Bjerke and Peterson 1994; Sommer et al. 1996) and absolute testicular weight (Bjerke and Peterson 1994; Mably et al. 1992a; Sommer et al. 1996), suggesting a potential effect of TCDD; however, relative testicular weights, when reported, were unchanged (Bjerke and Peterson 1994; Ohsako et al. 2002; Sommer et al. 1996), indicating that the testes were unaffected. Circulating levels of follicle-stimulating hormone (FSH), luteinizing hormone, and testosterone were unchanged (Bjerke and Peterson 1994; Mably et al. 1992a; Ohsako et al. 2001), suggesting that the hypothalamic–pituitary–testicular axis was also unaffected by TCDD. Morphologic assessment of the testes also failed to demonstrate evidence of treatment-related effects. In one study, testicular atrophy and separation of the caput and caudal regions with a loss of corpus epididymis was observed in two rats, which were excluded from the analysis (Ohsako et al. 2001).

Other than abstinence, Sertoli cell number is a central determinant of sperm count (Sharpe et al. 2003). In humans, Sertoli cell proliferation takes place during fetal, postnatal (0–8 months of age), and prepubertal development. Animal studies in mice and rats reveal that androgens primarily regulate Sertoli cell proliferation during the perinatal and prepubertal periods (Atanassova et al. 2005; De Gendt et al. 2004; Tan et al. 2005a, 2005b), whereas FSH plays a more central role in the peripubertal period (Johnston et al. 2004). Direct effects of developmental exposure to TCDD on the spermatocyte to Sertoli cell ratios were measured in adult Holtzman rats exposed to TCDD on GD15 (Mably et al. 1992a). TCDD did not affect the spermatocyte to Sertoli cell ratio at any age (PNDs 49, 63, or 120), leading to speculation of a defect in germ cell division or an increase in apoptosis of germ cells. Morphologic changes to the testis have been demonstrated in adult rats exposed to a single injection of either 3.0 or 5.0 μg TCDD/kg BW, whereas doses of 0.5 and 1.0 μg TCDD/kg BW had no effect on the testis (Chahoud et al. 1992). In that study, the number of spermatids per testis was decreased by TCDD treatment, and the spaces between adjacent Sertoli cells were enlarged, indicating dissolution of the germinal epithelium. Similarly, contacts between Sertoli cells and spermatogonia were disrupted in a subchronic study in rats treated with an initial dose of 25 or 75 μg TCDD/kg BW followed by a once-weekly maintenance dose of either 5 or 15 μg/kg, respectively, for 10 weeks (Chahoud et al. 1989). However, Sertoli cell changes were only seen at high doses, which are well beyond the concentrations reported to induce changes in DSP and epididymal sperm counts (Mably et al. 1992a). Thus, it is unlikely that TCDD-induced changes in sperm counts can be attributed to changes in Sertoli cell structure or function. Therefore, we propose that the primary effect of TCDD is not on the testes or on spermatogenesis. We postulate that adverse effects of TCDD are more likely related to developmental abnormalities of the reproductive tract and epididymal structure and/or function.

## Alternative Modes of Dioxin Action

TCDD-induced changes in cauda epididymal sperm counts and decreased weights of androgen target tissues such as the seminal vesicle, epididymides, and prostate provide clear evidence of developmental toxicity. The mechanism(s) of action underlying these responses remains unclear. Modes and/or mechanisms of action of dioxin-induced changes in cauda epididymal sperm counts and reproductive tract structure and function of potential relevance to human health include dysregulation of androgen signaling and enhanced phagocytosis of germ cells (reviewed by Phillips and Tanphaichitr 2008) and enhanced epididymal sperm transit.

### Dysregulation of androgen signaling

Circulating levels of testosterone and dihydrotestosterone (DHT) were decreased in adult rats exposed to high concentrations of TCDD in both time-course (15 μg TCDD/kg BW) and dose–response (100 μg TCDD/kg BW) studies (Moore et al. 1985). Similarly, serum testosterone concentrations were significantly reduced in adult male rats treated with 25.0 μg TCDD/kg BW and sacrificed 4 weeks later (Johnson et al. 1992). However, the concentration of TCDD needed to induce a change in circulating testosterone levels is very high relative to the doses that alter sperm counts and those documented in human exposure. Decreased seminal vesicle and epididymal weights corroborated the reduced circulating testosterone levels; however, DSP or testis weights were unaffected, suggesting that TCDD may have affected Leydig cell function, and morphologic assessment of the testes revealed a decrease in Leydig cell volume. In three different rat strains bred from TCDD-resistant and -sensitive strains, circulating levels of testosterone were decreased 17 days after treatment with a single oral dose of 1,000 μg TCDD/kg BW (Simanainen et al. 2004a). Similarly, *in utero* exposure to the highest dose of TCDD (an initial dose of 300 ng TCDD/kg BW followed by a maintenance dose of 60 ng TCDD/kg BW for the 2 weeks before mating) induced a 50% decrease in plasma testosterone levels (Faqi et al. 1998). Mably et al. (1992c) found a 60% decrease in plasma testosterone and a 41% reduction in DHT after *in utero* exposure to a single dose of TCDD, which although substantial, was not significant, whereas other investigators were unable to find treatment-induced changes in circulating androgen levels (Gray et al. 1995; Loeffler and Peterson 1999; Roman et al. 1995). In contrast, 1 μg TCDD/kg BW administered on GD15 increased circulating levels of testosterone on PND70 in three different rat strains with different sensitivities to TCDD (Simanainen et al. 2004b). No differences in circulating levels of FSH or testosterone were documented in adult Holtzman rats exposed to TCDD on GD15 (Mably et al. 1992a). Therefore, the reduction in sperm counts reported in animal studies is unlikely the consequence of TCDD-induced changes in serum androgen levels (Simanainen et al. 2004a, 2004b) or effects on Leydig cell structure or function.

Dysregulation of testosterone signaling has been documented in several animal studies but not detected in others. TCDD treatment induced a decrease in androgen receptor (AR) gene expression in ventral prostate and decreased weight, both evidence of decreased androgen signaling (Ohsako et al. 2001). Morrow et al. (2004) reported that activation of the AhR in LnCAP prostate cells by TCDD could inhibit androgen-dependent proliferation, which was mediated by cross-talk between the AhR and the AR. Transcriptional regulation of AR signaling (Kollara and Brown 2006) by competition between the AR and the AhR for nuclear transcription factors is another possibility (Kollara and Brown 2006). Competition between the AhR and AR for transcription factors has been documented in breast cancer cell lines (Kollara and Brown 2006); however, although intriguing, its relevance to the testis and human cells *in vivo* and during development of the male reproductive tract remains unknown.

### Dysregulation of epididymal function

Reports that a single TCDD injection on GD15 results in decreased epididymal or cauda epididymal sperm counts without effect on testicular DSP are difficult to reconcile. We postulate that the adverse effects of TCDD are mediated via changes in epididymal function as opposed to effects on the testes. Changes in sperm transit through the excurrent duct system or increased removal of damaged sperm from the epididymis could provide reasonable alternative explanations to TCDD effects on spermatogenesis. Several laboratories have examined TCDD treatment on sperm transport through the epididymides with divergent results. High-dose *in utero* and lactational exposure to TCDD (an initial dose of 25–300 ng TCDD/kg followed by 5–60 ng TCDD/kg throughout premating, mating, pregnancy, and lactation) decreased the time for sperm to transit through the epididymides in Wistar rats (Faqi et al. 1998). In contrast, exposure to 1.0 μg TCDD/kg on GD15 had no effect on sperm transit in Holtzman rats, suggesting that phagocytosis of sperm in the epididymides is increased (Sommer et al. 1996). Exposure of C57BL/6 mice to TCDD (0.1–50 μg TCDD/kg) for 24 hr resulted in a decrease in spermatozoan mitochondrial membrane potential compared with vehicle-treated control mice (Fisher et al. 2005). The effect was not evident in AhR-knockout mice, demonstrating that the effect requires activation of the AhR. However, there was no increase in the number of apoptotic germ cells in the testis and no change in morphology of the testis and epididymis.

In general, phagocytosis of spermatozoa by the epithelial cells of the cauda epididymidis is very low. It has previously been suggested that damaged spermatozoal cells can be phagocytosed (Sutovsky et al. 2001), but the interpretation of these data has been challenged (Cooper et al. 2002). Furthermore, if TCDD treatment did stimulate phagocytosis to the extent that sperm counts decreased by almost 40%, one would expect this to be obvious when examining the morphology of the epididymis. This does not appear to be the case, given the lack of reference of such an effect. A second possibility is that the blood–epididymal barrier is compromised, resulting in activation of the immune system, thus allowing macrophages to readily enter the lumen of the epididymis and attack maturing spermatozoa (Cyr et al. 2007). Although this may explain the loss of epididymal spermatozoa, we found no reports of large numbers of macrophages within the epididymal lumen. A more likely possibility is that spermatozoa transit time is altered, resulting in fewer spermatozoa stored in the cauda epididymis. The epididymis is a long, open-ended, and highly convoluted tubule (Robaire et al. 2006). Spermatozoa are stored in the cauda epididymidis, but the retention of spermatozoa in this region is not well understood. Perhaps the best explanation is related to the length and high degree of convolution in this region of the epididymis. Changes in the convoluted nature of the cauda or the length of the cauda could directly affect the quantity of spermatozoa retained in the epididymis. Antiandrogenic compounds such as phthalates reportedly alter epididymal development and coiling of the epididymal tubule (Barlow and Foster 2003), and flutamide significantly reduces the size of the cauda epididymis (McKinnell et al. 2000); however, whether the length of the epididymal tubule is also reduced is unknown. Wilker et al. (1996) reported that neonatal administration of TCDD resulted in a loss of epididymal segmentation, although they did not report whether this is associated with alterations in the cauda. Epididymal sperm transit time may also be affected by changes in the composition of epididymal fluid. Water is reabsorbed from the seminal fluid in the efferent ducts between the testis and epididymis (Hess 2002), a process regulated by estradiol and mediated by estrogen receptor-α (ERα) (Zhou et al. 2001). TCDD is known to block estrogen action by activating the AhR, which has been shown to partly bind to the estrogen response element of estrogen-dependent genes (Safe et al. 1998). In the epididymis, after treatment with antiestrogens, or in ERα-knockout mice, there is retention of water in the seminal fluid (Zhou et al. 2001), which is associated with a decrease in cauda epididymal sperm counts (Ruz et al. 2006). Less concentrated sperm would exhibit faster transit time, particularly in the cauda epididymis, because fluid pressure in the epididymal lumen would likely be increased. Thus, the effect of dioxin and dioxin-like chemicals on epididymal structure and function requires further study.

## Epidemiology

Semen quality and sperm counts have reportedly declined approximately 2% per year for about 50 years (Carlsen et al. 1992). That provocative review (Carlsen et al. 1992) led to an explosion of studies, some of which reported a decline in semen quality (Auger et al. 1995; Irvine et al. 1996; Younglai et al. 1998) and some of which did not (Pal et al. 2006). Although the reported decline in sperm counts remains controversial, there appears to be consensus for regional differences in semen quality (Jorgensen et al. 2001, 2002, 2006). Geographic differences in semen quality may also be accounted for by differences in race, genetic background, lifestyle, diet, and exposure to environmental toxicants.

Environmental contaminants have received growing attention, in part because animal studies have shown that some chemicals reduce sperm counts and because human exposure to environmental pollutants is potentially modifiable. Reduced sperm counts have been documented in studies of men occupationally exposed to different environmental contaminants (De Celis et al. 2000; Eskenazi et al. 1991), but the relationship between dioxin exposure and semen quality remains ambiguous. No difference in semen quality was found in Taiwanese men exposed prenatally to rice oil contaminated with PCBs and polychlorinated dibenzofurans (Yu-Cheng accidental exposure), despite the occurrence of chloracne—clear evidence of AhR activation (Guo et al. 2000). However, sperm concentration and sperm motility were decreased in dioxin-exposed young healthy men from Belgium, a region with high dioxin contamination (Van Waeleghem et al. 1996), and total sperm count was decreased in Belgian men from Flanders (Comhaire et al. 2007). In 101 Flemish men 5 months after exposure to PCB- and dioxin-contaminated food, Dhooge et al. (2006) observed that semen volume was decreased but sperm concentration was increased with an increase in dioxin-like activity (CALUX assay). The increased sperm concentration can be explained by decreased semen volume in these men, whereas total and free testosterone levels were also decreased with increasing dioxin-like activity. In contrast, no relationship between dioxin-like activity and semen parameters was found in Inuit men and men from three European populations with tissue residue levels representative of background exposure (Toft et al. 2007).

It is difficult to compare studies because of differences in reporting results, such as sperm count versus sperm concentration, methods of quantifying dioxin exposure, and differences in controlling for confounding factors such as age, smoking history, influence of mothers smoking during pregnancy, time from last ejaculation, use of medications known to affect semen quality, and health status of the study subjects. Another factor that may be important is the subject’s age at time of exposure, as shown in a recent study of semen quality in men acutely exposed to high levels of TCDD in Seveso, Italy (Mocarelli et al. 2008). In that study, men were stratified on the basis of their age at the time of exposure; men who were adults at the time of exposure showed no effects, while sperm counts were increased in men exposed during the peripubertal period. However, decreased sperm concentrations were observed in men who were between 1 and 9 years of age at the time of exposure (mean age, 6.2 years). Mocarelli et al. (2008) also reported a decreased percentage of motile sperm and of progressively motile sperm, without any effect on circulating testosterone levels, suggesting a critical time window for dioxin exposure during which the developing reproductive tract is more sensitive; this is consistent with the animal literature. In contrast to the animal literature, these human exposures were associated with a decrease in semen characteristics, especially motility. Taken together, epidemiologic data suggest that dioxin exposure affects the function of sex glands with a decreased semen volume but with no change in sperm count; these data are, in part, consistent with the animal literature but contradictory to a definitive effect of dioxin on spermatogenesis. Moreover, although preliminary, the epidemiologic data also suggest that concentration and developmental stage at the time of exposure may be an important determinant of dioxin effects on semen quality in the human population.

## Summary and Conclusions

Developmental exposure to TCDD has been fairly consistently linked with decreased cauda epididymal and ejaculatory sperm counts in animal studies, although at higher dose levels than those used by Mably et al. (1992a). Indeed, strengths of the animal literature are the demonstration of adverse effects on cauda epididymal sperm counts in multiple rodent species. The main weaknesses include different methods used to quantify sperm count; the paucity of studies that have examined TCDD effects on DSP and the ambiguous nature of those results; the absence of documented changes in testicular and spermatozoa morphology; and the divergence of effects of TCDD upon sperm counts and circulating androgen levels. Therefore, we find no credible evidence to support the conclusion that TCDD adversely affects spermatogenesis, with the exception of a small number of male rats in which testicular and epididymal lesions have been described (Ohsako et al. 2001). Alternatively, we suggest that effects of TCDD on androgen signaling, reproductive organ weights, and sperm transit through the epididymides are more plausible potential explanations for the reported decrease in epididymal sperm counts.
